# Supplementary material for: Single-cell reference mapping to construct and extend cell-type hierarchies
Source: NAR Genom Bioinform. 2023 Jul 26;5(3):lqad070. doi: 10.1093/nargab/lqad070 (PMC10370450; doi:10.1093/nargab/lqad070)
Supplement: lqad070_Supplemental_File [file lqad070_supplemental_file.pdf]

Supplementary Material

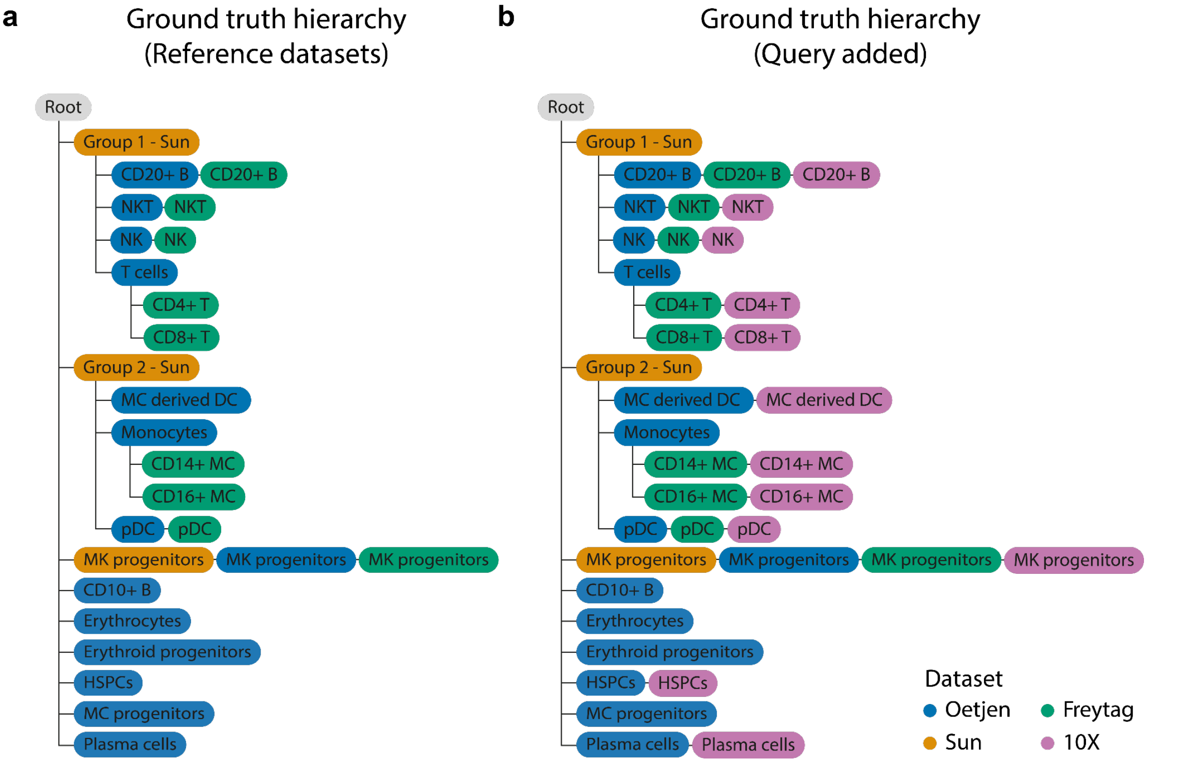

Figure S1: Ground truth hierarchy for a) the reference datasets (Sun, Oetjen, and Freytag) and b) when adding the query dataset (10X).

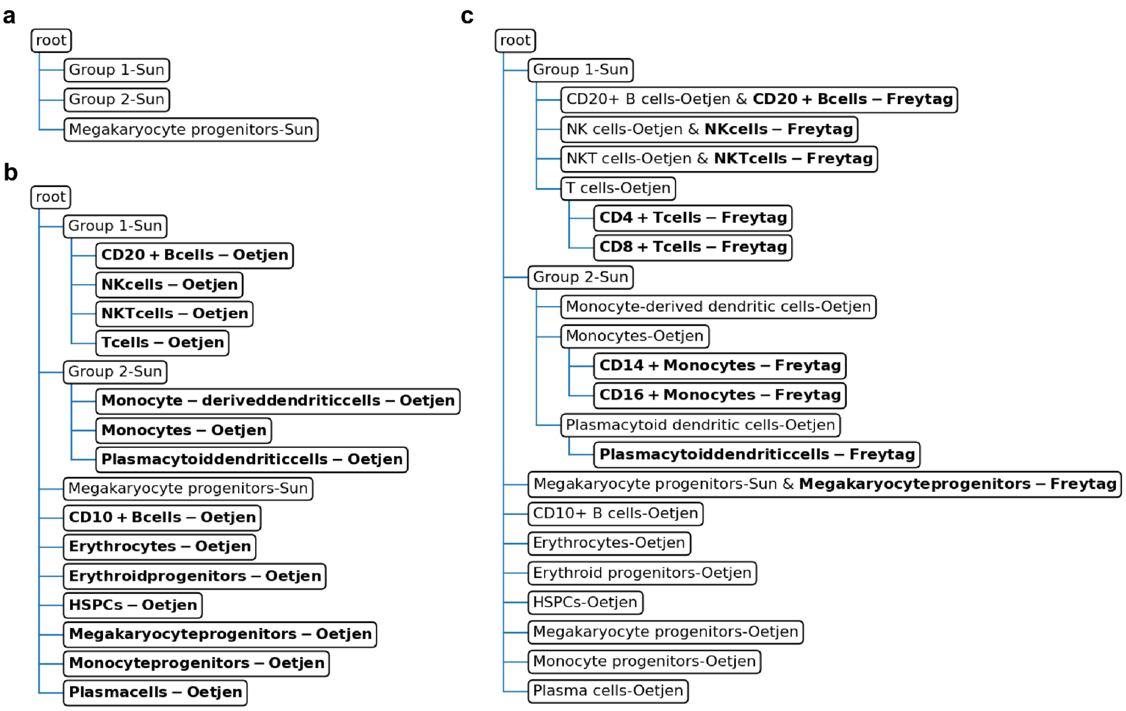

Figure S2: Intermediate step when creating the cell-type hierarchy for the reference PBMC datasets. a) Starting tree, which is a flat tree containing only the cell types of the Freytag dataset, b) Oetjen dataset added, c) Sun dataset added

a

b

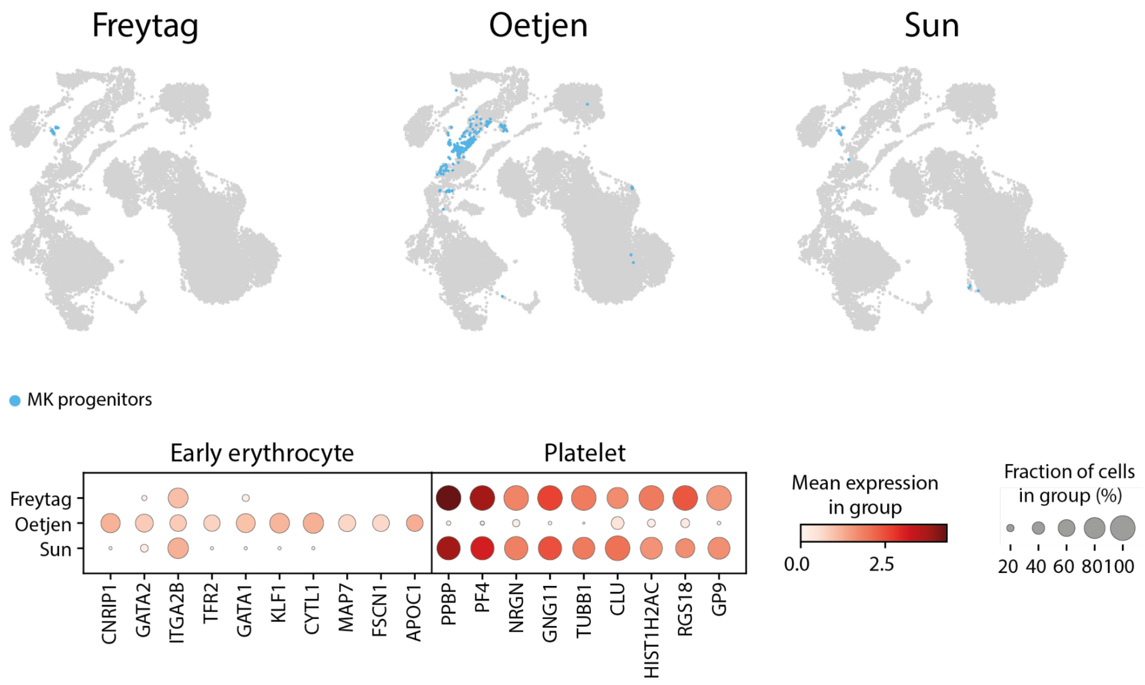

Figure S3: a) UMAP embedding showing the different cell types in the Freytag, Oetjen, and Sun dataset. Megakaryocyte (MK) progenitor cells of the Freytag and Sun dataset are at a different location than the Oetjen dataset. b) Marker gene expression for early erythrocytes and platelets in the three datasets.

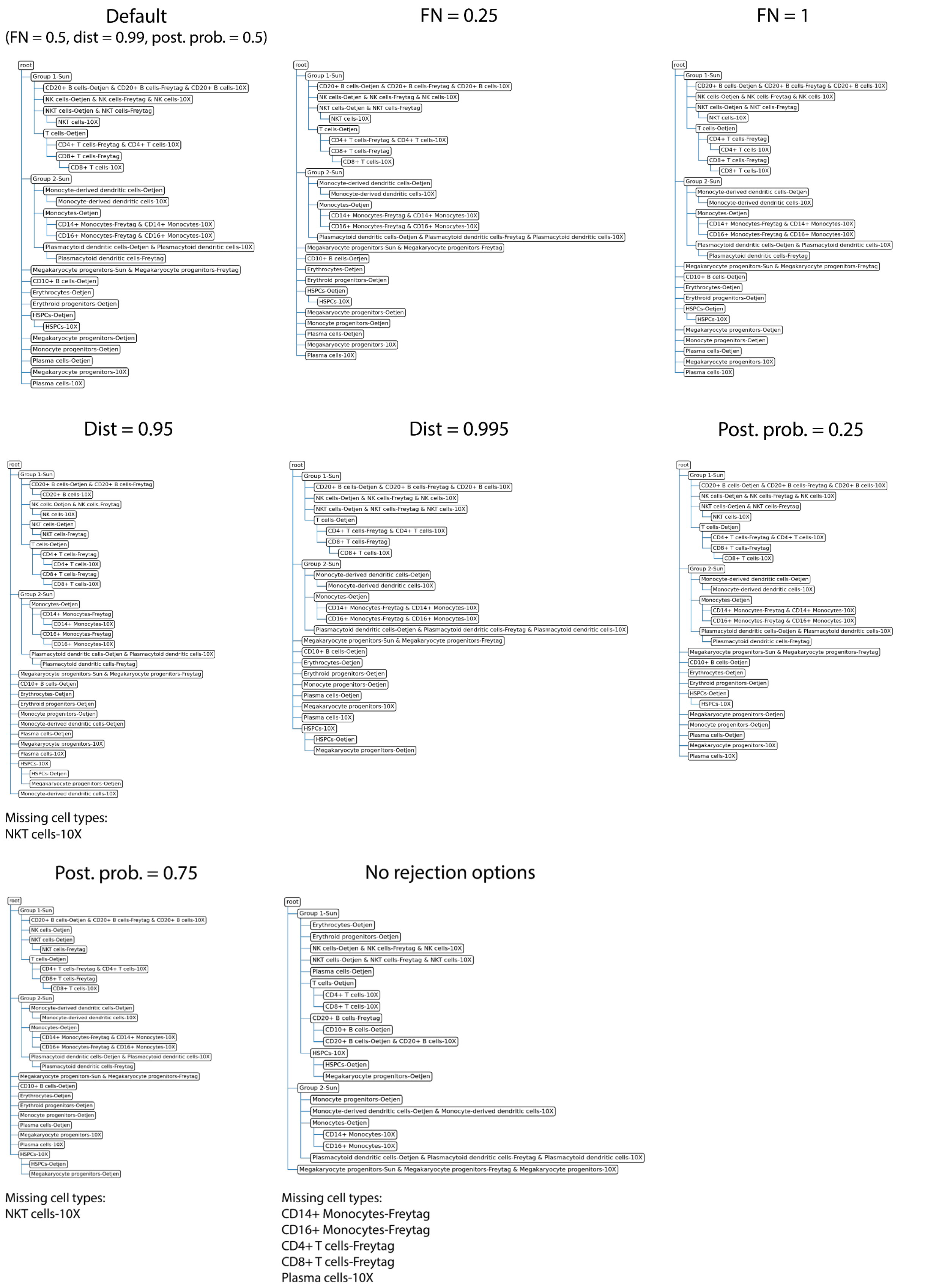

Figure S4: Constructed cell-type hierarchies for the PBMC datasets using different parameters for the three rejection options.

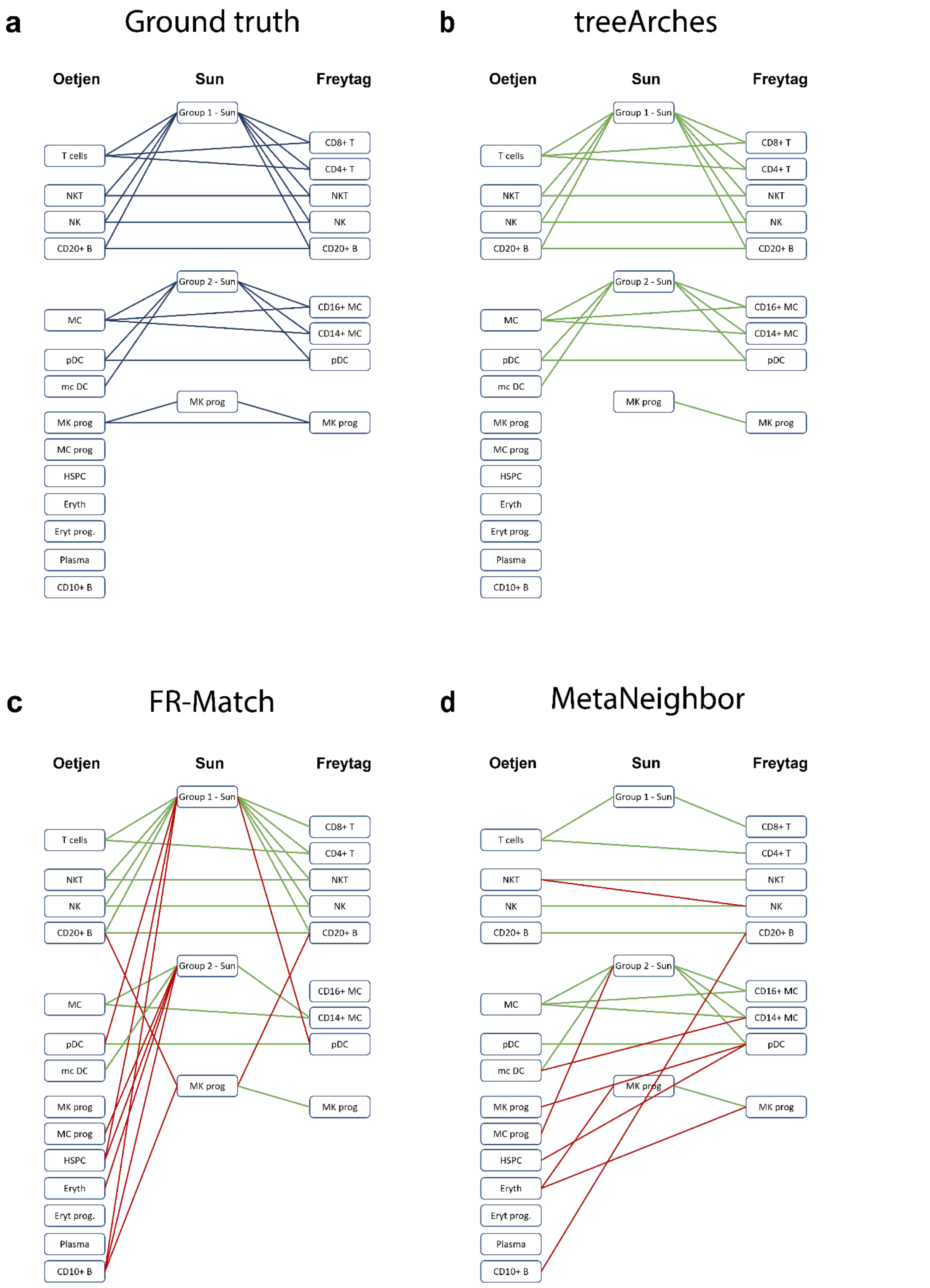

Figure S5: Comparison of cell type matching algorithms. a) Graph showing the ground truth matches between cell types. b-d) Results of treeArches, FR-Match, and MetaNeighbor respectively. Edges in green indicate a right edge, and edges in red indicate a wrong edge.

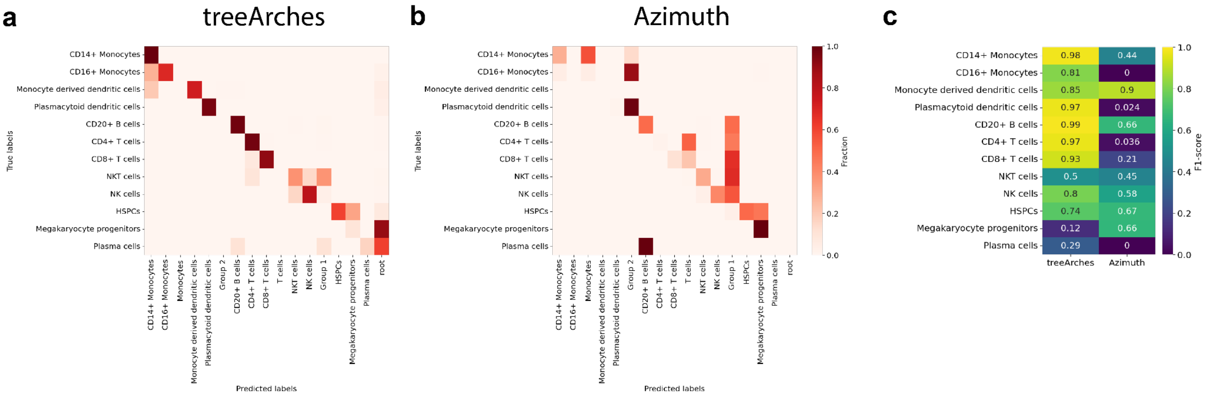

Figure S6: Comparison of classification performance using hierarchical labels. a-b) Confusion matrix for treeArches and Azimuth. c) F1 scores per cell type.

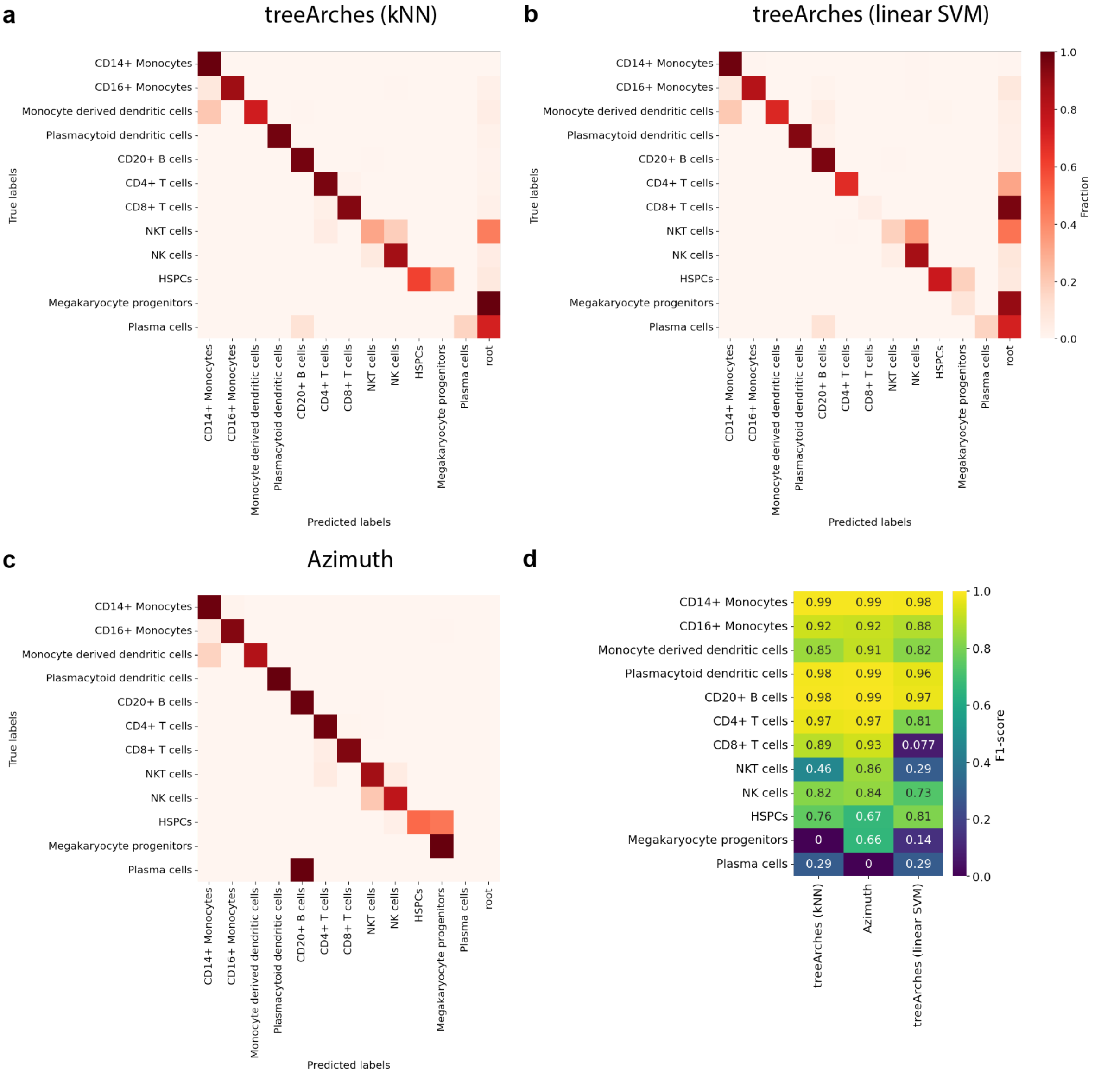

Figure S7: Comparison of classification performance using the harmonized labels. a-c) Confusion matrix for treeArches with kNN, treeArches with linear SVM, and Azimuth. d) F1 scores per cell type.

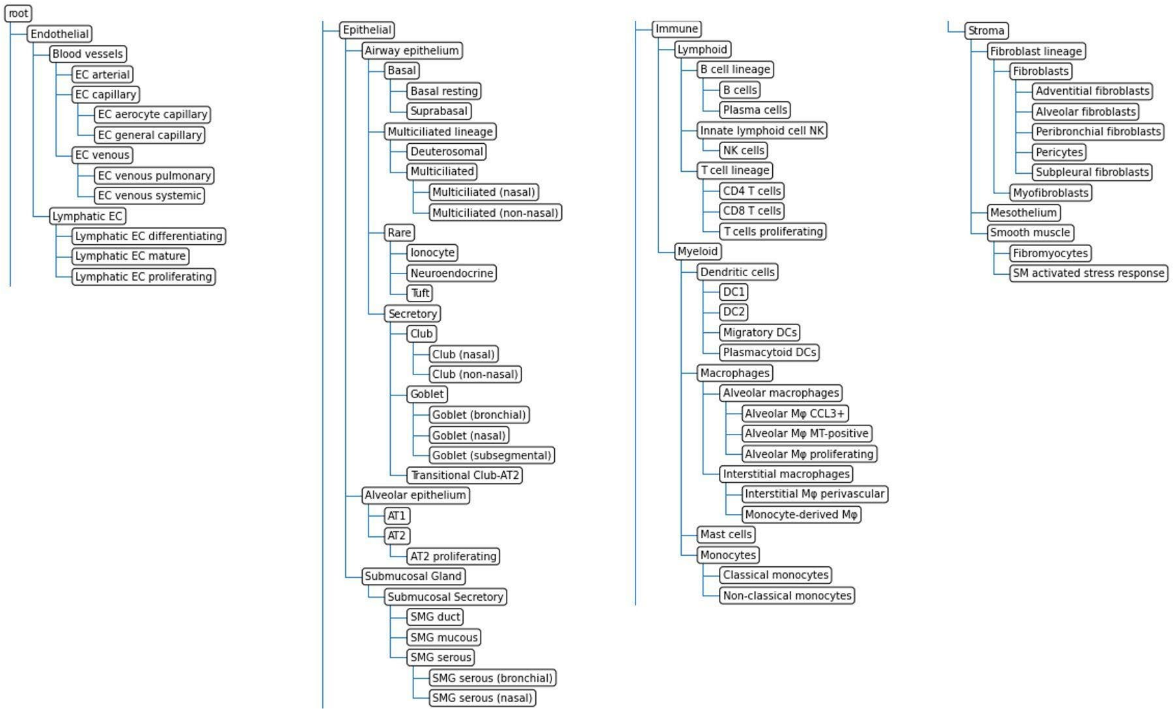

Figure S8: cell-type hierarchy constructed for the reference atlas (2).

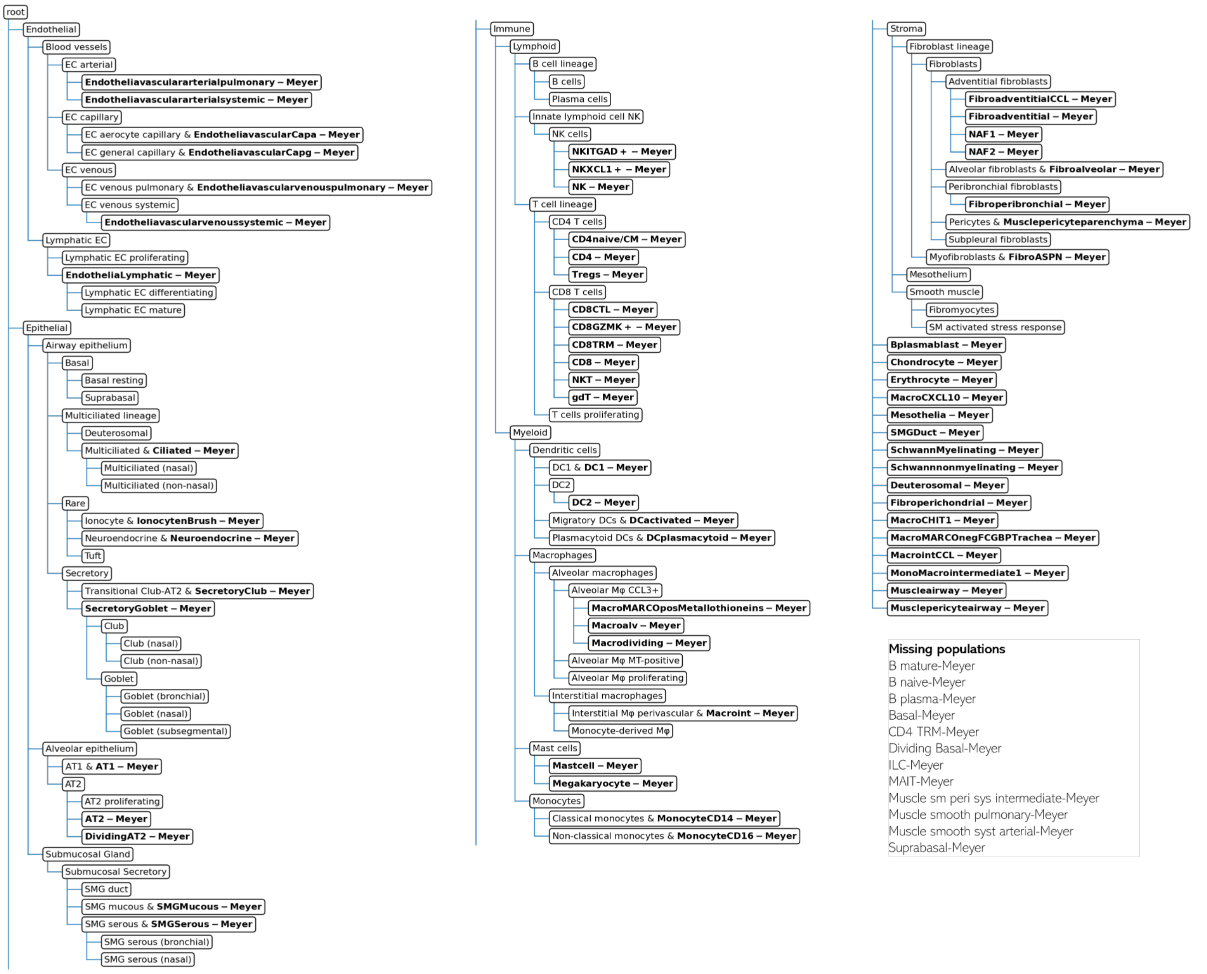

Figure S9: Updated cell-type hierarchy learned by adding a query dataset (Meyer dataset) to the reference tree.

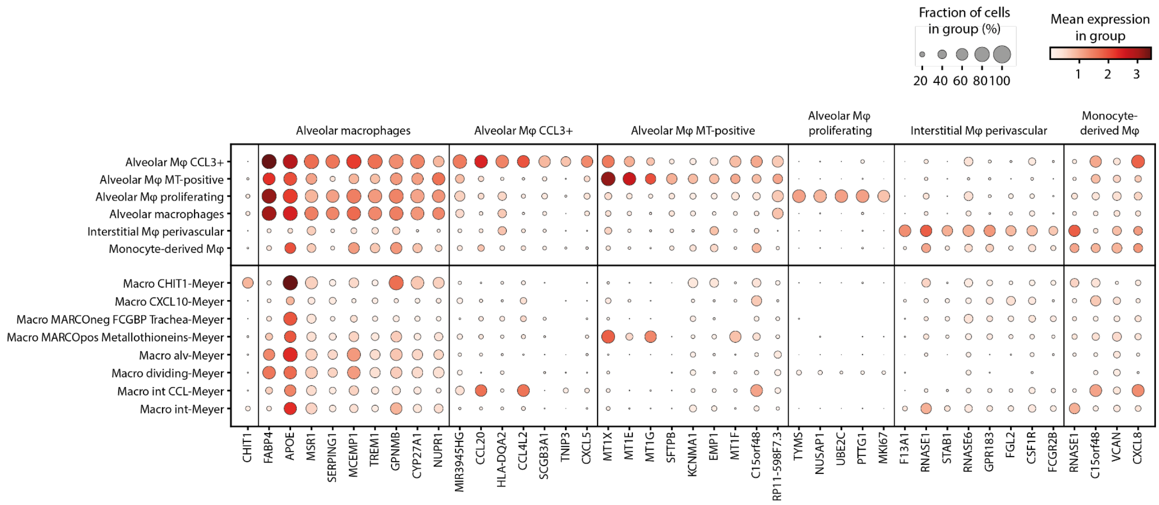

Figure S10: Marker gene expression for macrophage cell types in the reference datasets and Meyer dataset. The first column shows the expression of *CHIT1*, a gene used to annotate the Macro CHIT1 cells in the Meyer dataset. The rest of the genes are grouped according to the cell type in the reference atlas they were used as a marker for.

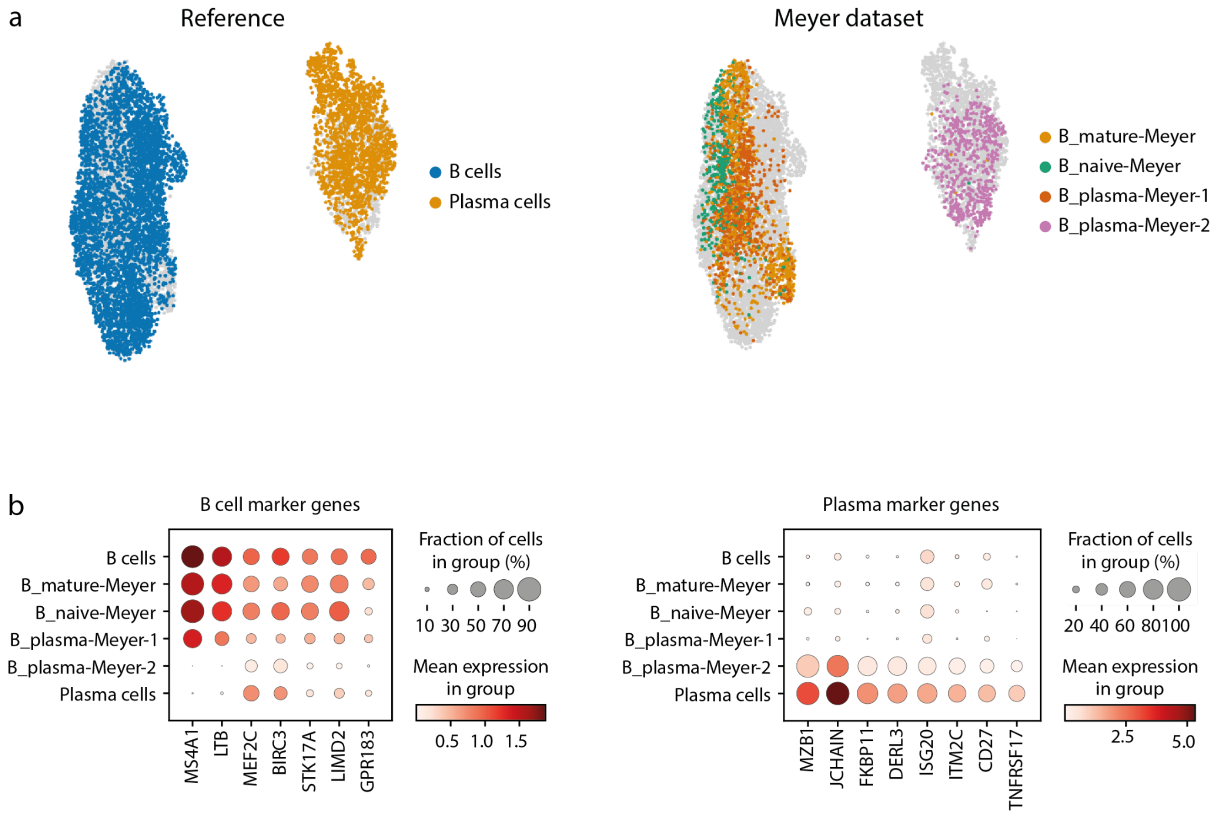

Figure S11: a) UMAPs showing the B cells and plasma cells in the reference and Meyer dataset. We split the plasma cells in the Meyer dataset into two groups. The first group overlaps with the reference B cells and the second group overlaps with the reference plasma cells. b) B cell and plasma cell marker gene expression in the reference and Meyer cell types. Plasma-1 from Meyer shows B cell marker gene expression, while Plasma-2 from Meyer shows plasma marker gene expression.

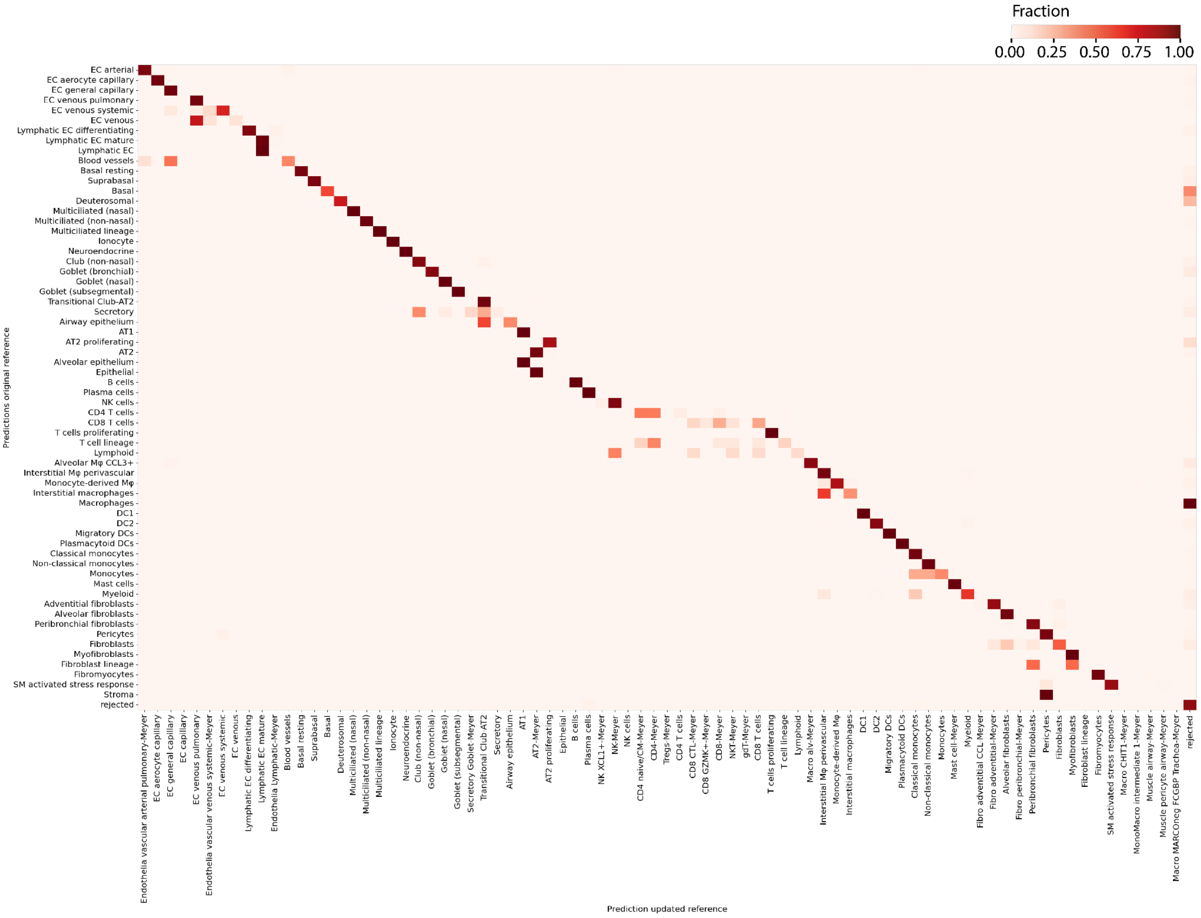

Figure S12: Confusion matrix comparing the predictions on the Tata dataset using the original reference and the updated reference.

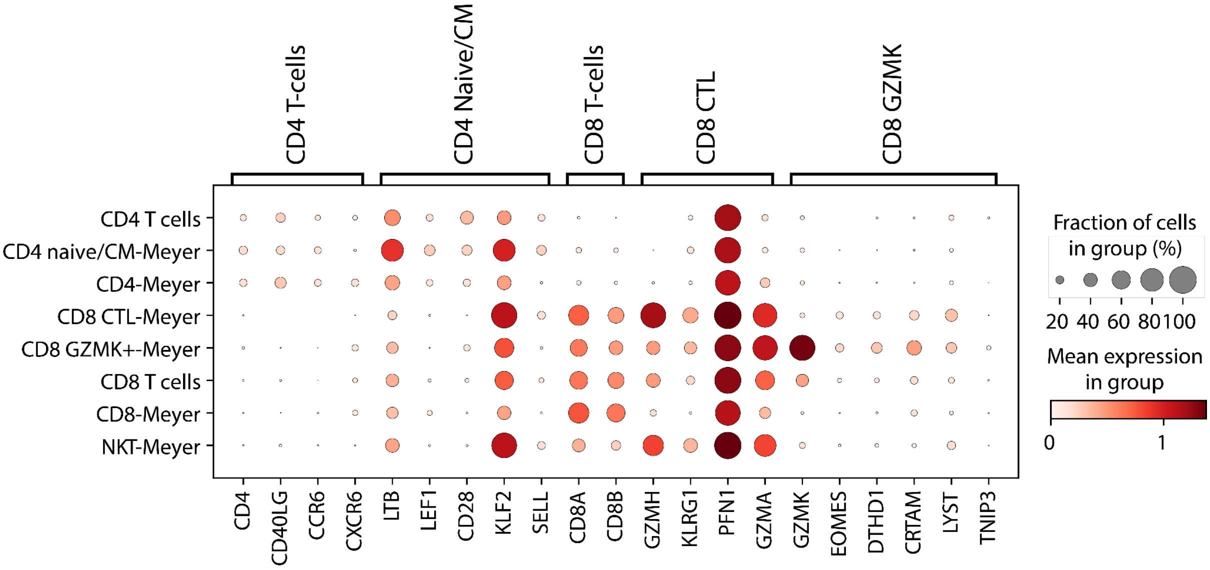

Figure S13: Expression of marker genes for CD4+ T cells, CD4+ naive/CM, CD8+ T cells, CD8+ CTL and CD8+ GZMK.

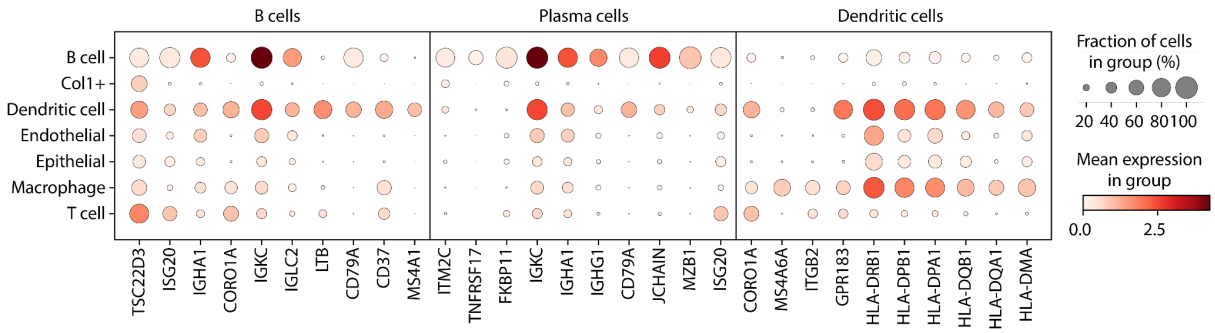

Figure S14: Expression of marker genes for B cells, plasma cells, and dendritic cells in the cell types in the IPF dataset.

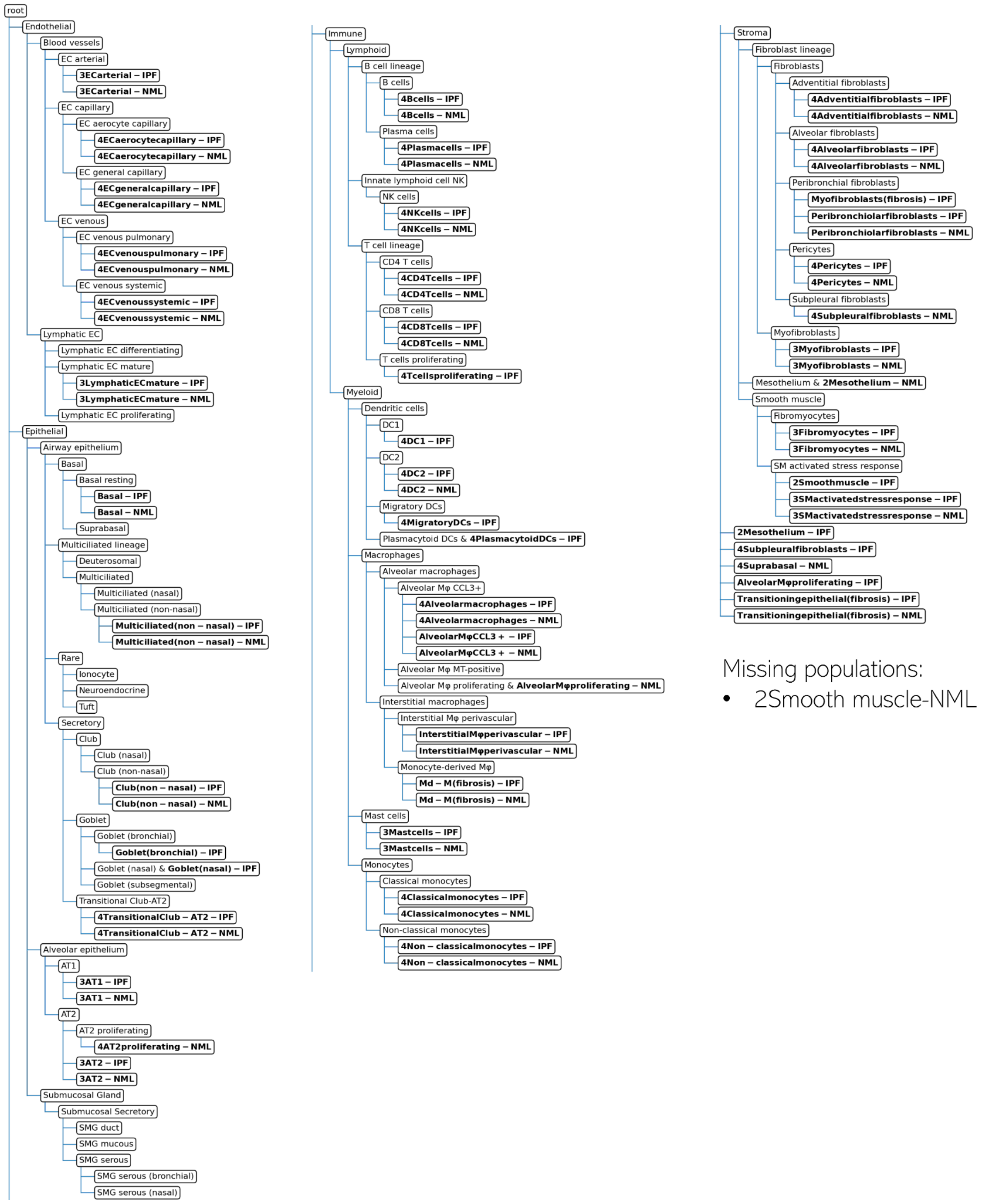

Missing populations:

- 2Smooth muscle-NML

Figure S15: Updated hierarchy of the HLCA after adding the IPF dataset (IPF condition and normal condition).

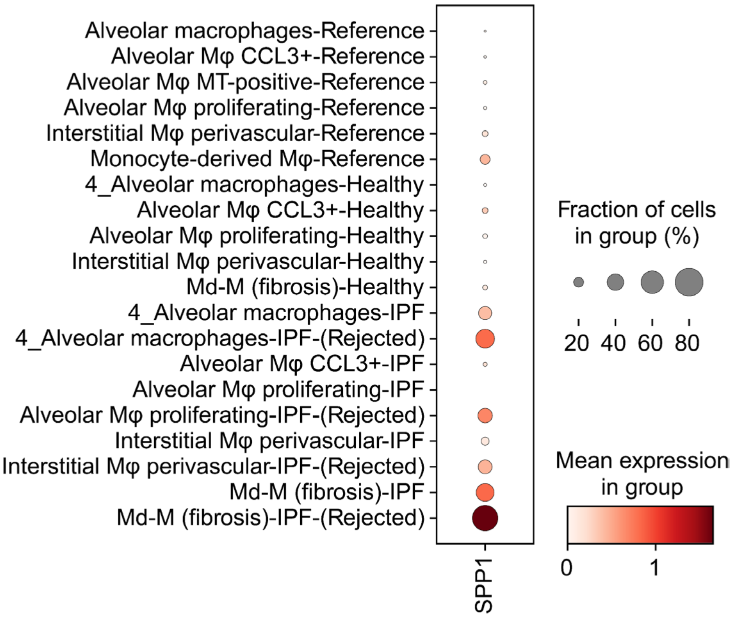

Figure S16: Expression of *SPP1* in the different reference and query cell types. The alveolar, interstitial, proliferating, and Md-M (fibrosis) IPF cell types are split into the rejected and non-rejected cells.

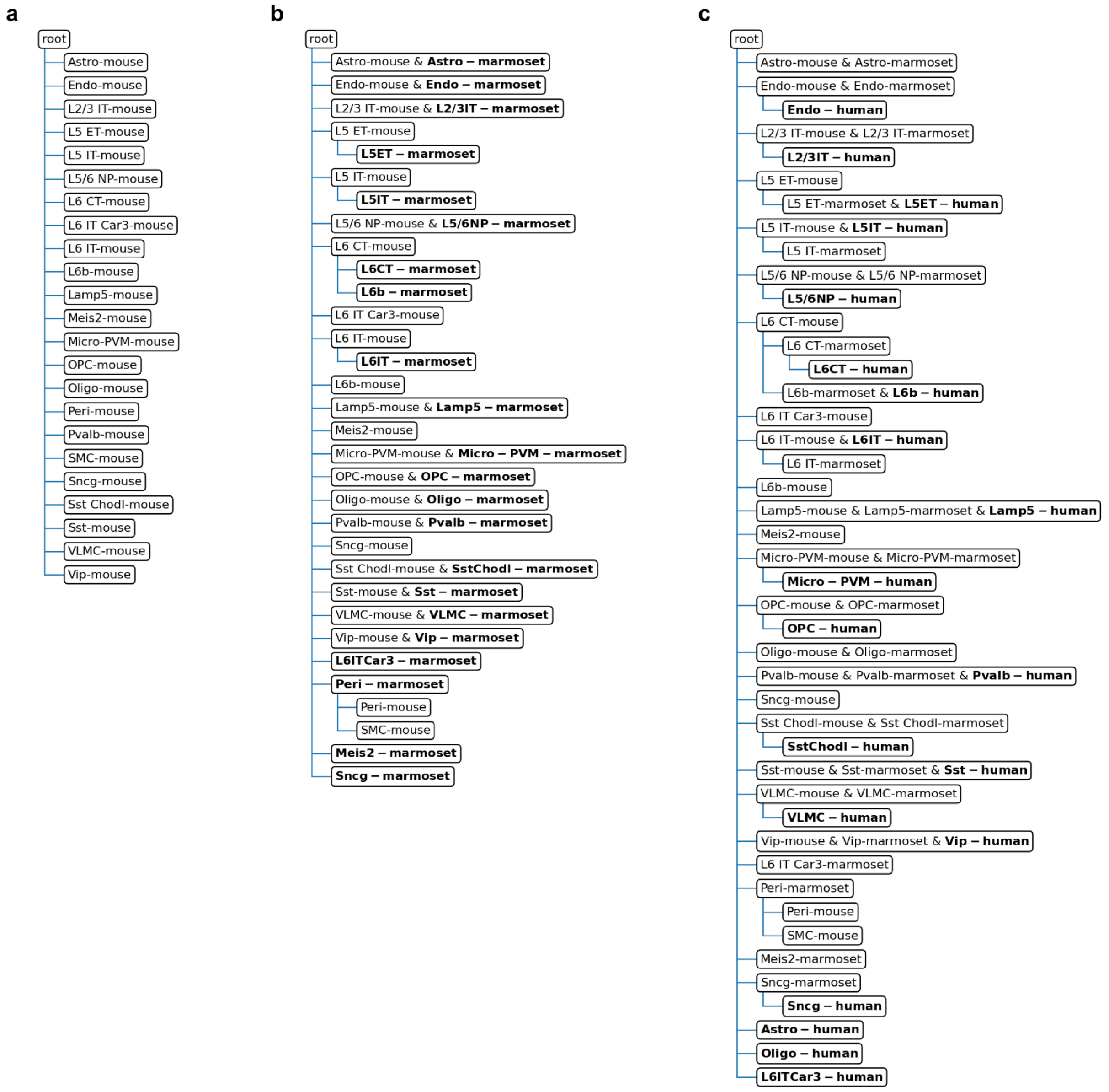

Figure S17: The complete hierarchy and intermediate steps when creating the cell-type hierarchy for the motor cortex datasets. a) Starting tree, which is a flat tree containing only the cell types of the moue dataset, b) marmoset dataset added, c) human added

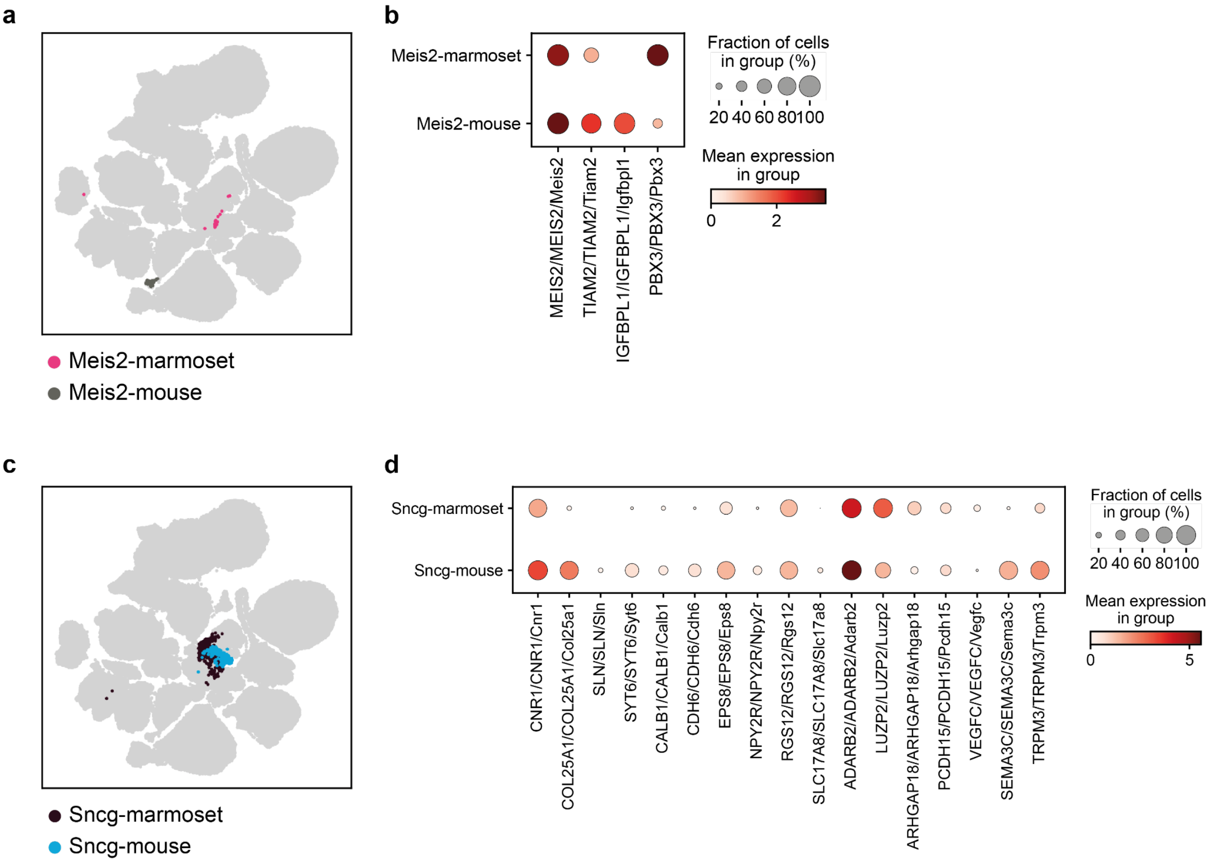

Figure S18: a) and c) UMAP embedding showing the integrated latent space of the reference datasets (mouse and human). The Meis2 and Sncg cell types are highlighted respectively. b) and d) Marker gene expression for the Meis2 and Sncg cell types respectively. The three gene names shown are the human/marmoset/mouse gene names.

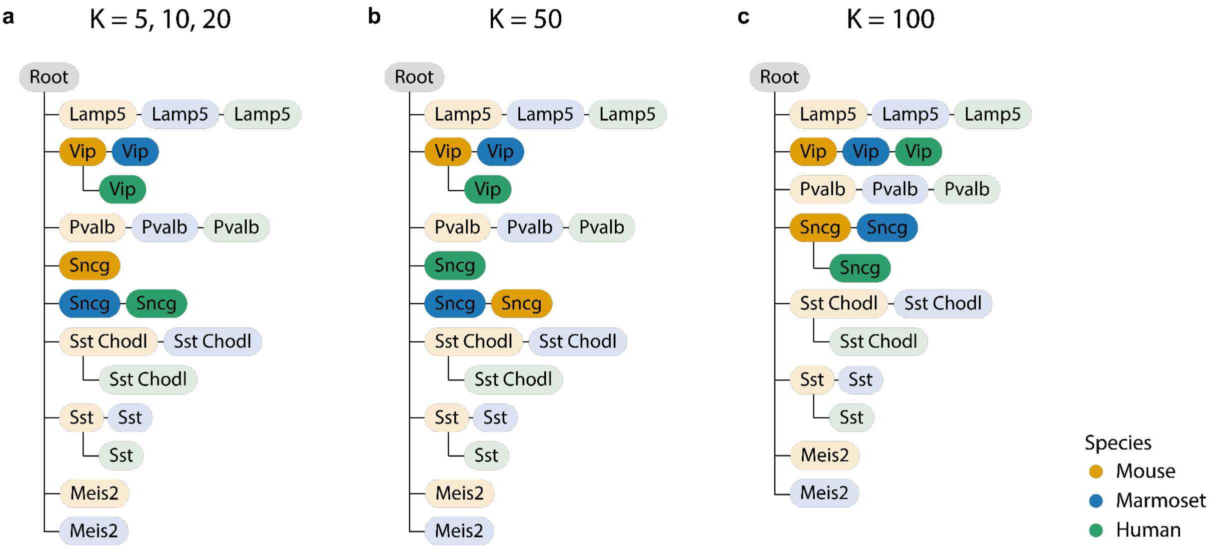

Figure S19: Influence of the number of neighbors (K) on the learned hierarchy. The nodes are colored according to the species they come from. The links between most nodes are robust and do not change when the number of neighbors varies. The differences between the trees are highlighted using brighter colors.

Table S1: Runtime and memory usage of treeArches on the different datasets. All runtimes are using 1 GPU.

	PBMC	HLCA (Meyer)	HLCA (IPF)	Cross-species
Total number of cells	32,484	713,512	646,487	305,638
Integrating reference	3 min	*	*	2 h
Constructing reference tree	0.5 min	*	*	22 min
Training reference tree	-	7 min	7 min	-
Integrating query	0.5 min	*	*	10 min
Updating tree with query	0.5 min	2.5 h	1.5 h	25 min
Memory	8.5 GB	6.3 GB	3.3 GB	81.9 GB

* For the HLCA, we used the latent space and hierarchy constructed in their original paper. As a consequence, we don't have to construct the hierarchy but only train the classifiers. This also explains the lower memory usage.

Table S2: Information on PBMC datasets used in this study

Dataset	Tissue	No. of Samples	No. of cells	No. of genes	No. of cell types	Protocol	Reference or query
Oetjen (19)	Bone marrow	3	9581	12303	16	10X v2	Reference
Sun (21)	PBMC	4	8829	12303	10	10X	Reference
Freytag (20)	PBMC	1	3347	12303	9	10X v2	Reference
10X (27)	PBMC	1	10727	12303	12	10X v3	Query

Table S3: Overview of cell types (original labels) in the PBMC datasets

cell type	Oetjen	Sun	Freytag	10X
CD4+ T	2524	4312	1238	2937
CD8+ T	985	578	270	350
NKT	608	649	432	1056
NK	89	973	476	756
CD20+ B	491	409	427	1546
CD10+ B	207			
CD14+ MC	997	1501	452	3388
CD16+ MC	165	271	25	364
MC derived DC	214	82		182
pDC	133	40	11	81
MK progenitor	219	14	16	21
Erythrocytes	1502			
Erythroid prog.	463			
HSPC	445			28
MC progenitor	428			
Plasma cell	111			18

Table S4: Cell type labels of the PBMC datasets after relabeling.

Original label	Oetjen	Sun	Freytag	10X
CD4+ T	T cells	Group 1 - Sun	CD4+ T	CD4+ T
CD8+ T	T cells	Group 1 - Sun	CD8+ T	CD8+ T
NKT	NKT	Group 1 - Sun	NKT	NKT
NK	NK	Group 1 - Sun	NK	NK
CD20+ B	CD20+ B	Group 1 - Sun	CD20+ B	CD20+ B
CD10+ B	CD10+ B			
CD14+ MC	MC	Group 2 - Sun	CD14+ MC	CD14+ MC
CD16+ MC	MC	Group 2 - Sun	CD16+ MC	CD16+ MC
MC derived DC	MC derived DC	Group 2 - Sun		MC derived DC
pDC	pDC	Group 2 - Sun	pDC	pDC
MK progenitor	MK progenitor	MK progenitor	MK progenitor	MK progenitor
Erythrocytes	Erythrocytes			
Erythroid prog.	Erythroid prog.			
HSPC	HSPC			HSPC
MC progenitor	MC progenitor			
Plasma cell	Plasma cell			Plasma cell

Table S5: Differentially expressed genes between the rejected and not-rejected cells

	Genes higher expressed in not-rejected cells	Genes higher expressed in rejected cells
NKT-cells (Oetjen)	*CD8A, CD8B*	*RGS1, ITM2A*
NKT-cells (Freytag)	*GLNY*	-
CD8+ T-cells (Freytag)	-	-
MC-derived DC	*ETV3*	*CKS1B, MKI67, RNASE2, TMPO, TK1, TYMS, ZWINT, DTYMK, UBE2C, CDKN3, BIRC5, HMGB3, CENPF, SMC2, CDC20, NUSAP1, TOP2A, CENPW, RNASEH2A, HIST1H4C, PTTG1, RRM2, SMC4, TROAP, PHF19, GGH, H2AFX, MCM7, NCAPD3, NCAPD2, MAD2L1, LIG1, CEP55, VRK1, GMNN, NUF2, CENPM, EZH2, PRC1, CDT1, RAD51AP1, ASF1B, TPX2, UBE2T, AURKB, SHCBP1, WDR34, FEN1, NCAPG2, CLSPN, PLK1, RPL39L, KIF11, KIFC1, CDCA7L, CENPK, NT5DC2, HIRIP3, LMNB2, CDK1, HMMR, PXMP2, BRCA2, SLC2A4RG, ACOT7, ASRGL1, CCNB2, GTSE1, CCNB1, ACAT2, TCF19, PHGDH, MND1, FOXM1, FANCI, CCNA2, RFC5, CDC25B, CENPE, ATAD3A, ATAD5, MYBL2, CDK5RAP2, CTNNAL1, GTF3C5*

Table S6: Quantitative evaluation of the cell type matching algorithms.

Method	Correct edges	Missing edges	Wrong edges
treeArches	24	2	0
FR-Match	19	7	11
MetaNeighbor	15	11	8

Table S7: Information on brain datasets used in this study

Species	No. of cells	No. of genes	No. of cell types (class/subclass/ RNA_cluster)	Protocol
Mouse	159,739	27,439	3/23/116	10X v3
Marmoset	69,279	27,466	3/22/94	10X v3
Human	76,621	32,991	3/20/127	10X v3
